# Exploring opportunities to enhance the quality of pharmacy-based contraceptive service delivery for adolescents and young women in Kenya: a multimethod qualitative study

**DOI:** 10.1186/s12913-025-12933-0

**Published:** 2025-06-03

**Authors:** Tessa Fujisaki, Dismas Congo Ouma, Merceline Awuor, Cellestine Aoko, Syovata Kimanthi, Elizabeth A. Bukusi, Elizabeth K. Harrington

**Affiliations:** 1 Department of Global Health, University of Washington, Seattle, WA USA; 2 Centre for Microbiology Research, Kenya Medical Research Institute, Kisumu, Kenya; 3 Centre for Clinical Research, Kenya Medical Research Institute, Nairobi, Kenya; 4 Department of Obstetrics & Gynecology, University of Washington, Seattle, WA USA

**Keywords:** Pharmacist, Family planning, Kenya, Contraception services, Adolescent girls and young women

## Abstract

**Introduction:**

Private-sector pharmacies are a preferred source of contraception in many low- and middle-income country settings, especially among adolescent girls and young women (AGYW), but the quality of contraceptive care and counseling in community pharmacies remains understudied. This study aims to identify and describe barriers, facilitators, and opportunities to improving the quality of pharmacy-based contraceptive care for AGYW.

**Methods:**

In this multi-method, cross-sectional study, conducted from March to June 2023, we triangulated data from semi-structured qualitative interviews with pharmacy providers, flow mapping, and AGYW mystery clients at 20 community pharmacies in Kisumu County, Kenya. Interview transcripts were iteratively coded and analyzed thematically by an interdisciplinary team. Flow maps were visually summarized, and mystery client evaluations were analyzed in a thematic matrix.

**Results:**

Pharmacy providers identified time, concurrent clients, space, lack of adequate provider training, lack of client health education, and stigma related to AGYW sexuality and contraceptive use as key barriers to quality pharmacy-based contraceptive counseling; facilitators included existing rapport with AGYW, provider motivation to serve marginalized AGYW, and respect for client privacy. Providers expressed high demand for training and job aids, and concerns about ineffective or overuse of emergency contraception among AGYW. Flow mapping and mystery client evaluations revealed a variety of quality gaps, including privacy and time bottlenecks, and lack of adequate sharing of contraceptive information.

**Conclusions:**

Our data suggest multiple avenues to improve contraceptive care quality through efforts to enhance privacy and counseling, with the potential for improved reach, person-centeredness, and ultimately reproductive health outcomes for youth. Future research is needed to find the balance between improving quality and maintaining the qualities that make pharmacies preferable to youth in various contexts.

**Supplementary Information:**

The online version contains supplementary material available at 10.1186/s12913-025-12933-0.

## Background

Private-sector pharmacies are a preferred source of contraception in many low- and middle-income country (LMIC) settings, especially among adolescent girls and young women (AGYW; age 15–24) [1]. An analysis including data from 33 LMIC countries in the World Health Organization African Region showed that half of contraceptive users aged 15–19 years and 20% of AGYW sourced their method from a pharmacy, drug shop, or informal provider, despite rating the quality of care more poorly than in formal clinics [2]. Young women’s preferences for the community-based pharmacies as access points for contraception are often driven by characteristics such as friendly, non-judgmental providers, speed of service, discreetness, proximity, and convenience [3–5], with a significant body of work conducted in Kenya [3, 6, 7]. While it is well-documented that AGYW living in LMICs face unique, multilevel barriers to pregnancy prevention – including internalized and community-level stigma, unequal power in sexual relationships, and lack of education and financial resources [8–11], many marginalized AGYW have identified the pharmacy as a lower-barrier contraceptive source. Furthermore, a variety of political and policy challenges to addressing adolescent sexual and reproductive health issues exist in Kenya [12], including conflicting laws and policies regarding adolescent consent for care [13, 14].

While pharmacies have a key role in enhancing the accessibility of shorter-acting and episodic methods [2], limited studies demonstrate that quality of contraceptive care in pharmacy settings is uneven and some elements of service delivery are inadequate or missing [6, 15–17]. The Bruce/Jain framework, originally published by Bruce in 1990 [18] and revised to center rights-based family planning in 2018 [19], has been widely used to conceptualize quality of care in contraceptive service delivery. The revised framework focuses on structural dimensions (choice of methods, availability of a trained/competent provider, availability of space for audio/visual privacy, and availability of other SRH services) and process dimensions (appropriate information exchange with clients and respectful interpersonal relations) [19]. Quality of care is also related to the person-centeredness of counseling and communication, reflecting how counseling responds to individuals’ values and preferences [20, 21].

Gaps in quality of care in community pharmacies are somewhat predictable, as one would not expect a small private pharmacy to be comparable in space, material resources, provider training and cadre, and public funding to a clinic facility. Furthermore, efforts to improve the quality of contraceptive service delivery for AGYW in pharmacies according to the Bruce/Jain framework ironically run the risk of compromising the very qualities that are attractive to youth: for example, if they introduced longer wait times, asked AGYW to answer sensitive questions, or documented in a formal medical record [1]. There is increasing interest in capacity-building interventions such as public-private partnerships and commodity management to improve overall contraceptive access and quality of pharmacy-based care [22], but the in-pharmacy opportunities to enhance the AGYW experience of contraceptive care and counseling have not been sufficiently explored.

In the current study, we aim to identify and describe barriers, facilitators, and opportunities to improving the quality of pharmacy-based contraceptive care specifically for AGYW in Kenya, with a focus on contraceptive counseling and information. This qualitative study triangulates semi-structured qualitative interviews with pharmacy staff, flow mapping, and AGYW mystery clients to gain balanced insight into potential strategies for quality improvement.

## Methods

### Study overview and setting

Data for this multi-method, cross-sectional study were collected between March-June 2023. This research was conducted in urban and peri-urban Kisumu County, a major urban center located in western Kenya on the shores of Lake Victoria. We situated the study in Kisumu given our collaborative team’s long-term research engagement with AGYW in the region around contraceptive decision-making, contraceptive preferences, and the social and community-level influences on contraceptive access and use, which provides context for the current study. Kisumu County is the cultural center of the Luo ethnic group and has a multi-ethnic population with diverse representation from other tribes. According to the 2022 Demographic and Health Survey, the median age at first birth in Kisumu County is 19 years (national average is 21 years), and the modern contraceptive prevalence rate among sexually active unmarried women aged 15–19 is 44% [23], with male condoms, emergency contraception (EC), injectables, and implants the most commonly used methods.

Pharmacies in Kenya, which are regulated by the Pharmacy and Poisons Board, a public entity, typically stock several contraceptive methods, including EC, oral contraceptives (OC), male condoms, and injectables. The National Family Planning Guidelines were amended in 2018, allowing pharmacists and pharmaceutical technologists to administer injectable contraception, which remains the most frequently used method in the country [24]. In Kisumu County, community retail pharmacies are abundant, with 119 licensed in 2022 [25]. In contrast to public sector clinics, many community pharmacies are open outside of typical business hours in the evening and on the weekend, enhancing convenience and accessibility for working and school-going individuals [3].

### Sampling and data collection

#### Qualitative interviews and flow mapping with pharmacy staff

We used purposive sampling to select 20 geographically dispersed community pharmacies in urban and peri-urban Kisumu. Quotas were established to ensure adequate representation from densely populated informal settlements and presence of a private consultation room. Eligible pharmacies provided OC, EC, condoms, and injectables, and had a pharmacy owner who supported study participation. The pharmacy owner designated themselves or an interested pharmacy staff member fluent in Kiswahili, Dholuo, or English to participate in a semi-structured interview, and written informed consent was obtained. Experienced trilingual qualitative research staff (MA, CA) conducted interviews in May-June 2023 on the pharmacy premises. The pre-piloted interview guides (see Supplemental Material) introduced dimensions of quality and covered topics such as contraceptive delivery practices, barriers and facilitators to providing contraceptive counseling for AGYW, perceived training needs, and perspectives on the utility of contraceptive decision support tools. The 45–60 min interviews were audio-recorded, transcribed verbatim by the interviewers, and translated into English where applicable.

Upon interview conclusion, study staff initiated data collection for flow mapping evaluations (see Supplemental Material). Pharmacy staff led interviewers through the physical space of the pharmacy, mirroring the journey of an AGYW client seeking contraception from arrival at the pharmacy through their departure after service provision. Interviewers asked a variety of probe questions about privacy and time bottlenecks in various circumstances; they took written notes and sketched out an annotated flow map to describe the data. Pharmacy staff participants received 1500 Kenyan Shillings (approximately USD$11–12) in appreciation of their time and expertise.

#### Mystery client evaluations

Mystery client methodology, in which study team members pretend to be a client or patient in a real-world health care setting and use standardized scripts to engage with a provider, is a well-established approach to collecting client experience and quality of care data [16, 26, 27]. We used published best practices [4] to develop training materials and detailed protocols to guide mystery client interactions in pharmacies. Mystery clients were 2 young women with roles as peer mobilizers for the USAID DREAMS program [28]. Both were 19 years of age, were fluent in Dholuo, Kiswahili, and English, were secondary school graduates, and resided in Kisumu. After attending a one-day training in study procedures and data collection, mystery clients posed as clients seeking contraceptive methods at the 20 previously selected pharmacies. Mystery clients were assigned a specific method to request at each pharmacy (OC, EC, or injectable), along with a specific question to ask the pharmacy provider (“How do I use it?”, “Does [method] have any side effects?”, [EC only] How often can I take this?”). They purchased a method at each pharmacy, including vials of injectable contraception, but declined to be injected at the pharmacy (it is a common practice to buy the drug only and be injected elsewhere). During their time at the pharmacy, mystery clients observed various aspects of the care experience, including the wait time to be served and total time at the pharmacy, method cost, perceived privacy, character of the interaction with pharmacy staff, any quality assurance steps prior to obtaining the method (e.g., asked about last menstrual period, recommended to do a pregnancy test, etc.), information volunteered by the pharmacy provider, and provider response to the assigned question. Immediately after leaving the pharmacy, mystery clients completed a written assessment, followed by same-day, in-person debriefings with study staff.

### Data analysis

The audio-recorded interviews were transcribed and translated into English where necessary by the study interviewers, anonymized, and imported into Dedoose qualitative data analysis software (version 9.2.22) for management and coding. The transcripts were thematically analyzed using an iterative, collaborative approach by 4 study team members. First, the analytic team developed an initial codebook based on key concepts of interest reflected in the interview guide (see Supplemental Material), and revised the codebook to include concepts emerging from the data after each analyst had coded 5 transcripts. All transcripts were coded by at least two analysts. The analytic team met weekly to review analytic progress, resolve coding discrepancies, and discuss evolving themes. After coding was completed, the members of the data analysis team prepared thematic code summaries to address primary research questions.

Flow mapping data and mystery client assessments were analyzed in tandem to develop a visual depiction of a flow map in Lucidchart, a web-based visual diagramming software program (www.lucidchart.com). Sketches of the physical layouts of the pharmacies were combined to create a ‘typical’ floorplan. Written notes from the flow mapping exercise and mystery client assessments were transcribed into a data matrix to summarize the process of obtaining contraceptive methods in the pharmacy setting, highlighting quality and time bottlenecks. The mystery client data matrix was then separately analyzed for themes, with an emphasis on perceived privacy, ease of communication with the pharmacy provider, and quality of counseling provided.

## Results

Among the 20 pharmacy providers who completed qualitative interviews, there were 10 women and 10 men. The majority (13/20) were trained as pharmaceutical technologists; 3 were pharmacists, and 4 were registered nurses. Over half (12/20) were aged 30–39 years, 5 were 20–29 years, and 3 were 40–49 years. All study pharmacies provided oral contraceptives (OC), emergency contraception (EC), male condoms, and injectable contraception; additionally, they all had access to a consultation room or store room separate from the public-facing counter.

Contraceptive implants were provided in 3 pharmacies that employed nurses or clinical officers.

### Provider perspectives

#### Barriers to quality pharmacy-based contraceptive care provision for AGYW

Pharmacy providers highlighted a variety of barriers to the provision of quality contraceptive care, including contraceptive counseling, for AGYW in their respective pharmacies. All pharmacy providers introduced barriers related to the pharmacy setting, which centered on time constraints from both the provider and client perspectives. Providers explained that pharmacies must operate as small businesses with modest margins, keeping staffing lean and selling as much product as possible. Depending on the circumstances related to client load and available staff, providers reported limited ability to answer method-related questions and offer individualized contraceptive counseling, especially during peak evening hours:

*“Time is…a challenge, the counseling requires more time, so that when you sit with the client you are able to explain to them all sorts of things that is there, but now you see if you take your time with 1 client, those who are at the counter will be impatient and might leave. They are also not paying for this session and you are losing customers on the other side. Although maybe the payment for the session might not be a problem, you might not have time because other people are also waiting for you at the counter and you are losing business.” (Nurse, female, age 30-39)*

Multiple providers noted that young women clients, especially school-going AGYW, were often the ones who were in a hurry, and frequently declined to receive counseling even as new contraceptive users. Providers directly related these time constraints to stigma and fear of being identified by known adults as seeking contraception or missed at home by family members who might ask them to account for their absence.

*“They [AGYW] are in a hurry—'give me P2 [EC]’—like for example…you are supposed to get time to talk to them, but they tell you, ‘there is my motorbike taxi waiting for me’. And you cannot force them. (Nurse, female, age 20-29)*

Some pharmacy providers also mentioned a lack of space and privacy as linked obstacles to comprehensive contraceptive services in the pharmacy. Not all retail pharmacies have a consultation room or a storeroom allowing for private space, and sometimes clients are served together at the counter and counseling can be overheard. Even if a consultation room is available, clients who are paying for services like HIV or malaria testing or who require injections are prioritized for the space. Participants described that when multiple clients are around, an adolescent girl might wait for everyone else to leave before asking for what she wants or leave before being served. Other AGYW may only be willing to be served by a female provider:

*“The level of privacy here is okay but the problem is that I'm the only female provider here at the moment…Right now we are three, one female and two males. You find that they're not free [open] with the male ones to an extent that they opt to wait for me if I’m not around because we work in shifts. If they happen to come when I’m still off duty, they are usually not free [open].” (Pharm tech, female, age 30-39 )*

Pharmacy providers strongly highlighted the role of stigma in shaping how AGYW engage in pharmacy-based contraceptive care. They explained that sexual activity and contraceptive use among young unmarried women are stigmatized in local communities, prompting AGYW to minimize time at the pharmacy and employ coded language or signals when requesting contraception to avoid detection by others, like holding out two fingers to signal the need for “P2”, which is the brand name of EC that is widely stocked in the region. Many providers reported taking contraceptives out of their packaging and placing them in plain brown paper bags on behalf of AGYW clients. Some AGYW even send younger siblings to procure contraceptives.

*“Actually for the young girls we have a tricky situation because within the community there are some people who have refused to accept that they are sexually active, so they tend to shy away from accessing it directly. So they can use their friends or the younger kids, they send the younger kids to access for them on their behalf since they don’t want any suspicion from the provider…and their parents too.” (Pharm tech, male, age 40-49)*

Most providers mentioned their own lack of formal training on contraception as a barrier to optimal care and contraceptive counseling, and expressed a desire for additional comprehensive training:

*“Many people haven't got that training concerning the family planning, so it becomes difficult whenever a client comes with hectic questions so sometimes you just do the guesswork. So whenever there is that training and the provider is certified, then I believe they can give out the best…to the client.” (Pharm tech, male, age 30-39)*

While providers generally referenced the “myths” AGYW believe about contraceptive harms, provider bias, and knowledge gaps also emerged as key barriers to quality contraceptive counseling. Several providers shared that they generally discouraged AGYW, especially those who have not yet given birth, to use certain or even all methods due to concern about “hormonal imbalance,” future infertility, and other health risks. Some declined to give certain methods and advised abstinence. As 1 pharmaceutical technologist explained,

*“Once she has given me the reason why she wants the method, from there I tell her the disadvantages of family planning methods. First, I will discourage her from using the method because they are not safe for those who have not given birth. They have a lot of issues and problems such that when she’ll be ready to have a child, she'll experience problems or health complications.” (Pharm tech, female, age 20-29)*

#### Facilitators and opportunities to improve contraceptive care and counseling

Providers reviewed multiple reasons why many AGYW choose pharmacies as their source of contraception, explaining how those reasons related to facilitators of quality pharmacy-based contraceptive care. First, they emphasized the key role of trust and provider rapport-building skills in interacting with AGYW seeking contraception. When providers are approachable and friendly, demonstrate respect for client privacy, and do not ask too many questions, AGYW are more likely to open up to them, allowing for meaningful interpersonal interaction and thus improved quality of counseling. Appropriate levels of privacy may be possible at the front counter or require a dedicated private space away from the public eye, depending on the individual interaction and presence of other clients. Many providers expressed compassion for the difficult life situations youth are experiencing, and named the need for providers to “embrace the circumstances they [AGYW] are in” without judgment:

*“The privacy part is very integral when it comes to giving these services because, these girls do not want to be judged, so privacy comes at the time when we offer these things to them, and giving them various options to choose from, we don’t judge them. So I can say that at that level we have done our level best because what we encounter with…[a client] remains between the two of us, yes that confidentiality first.” (Pharm tech, male, age 30–39)*

Pharmacy providers also mentioned adequate stock of methods AGYW tend to seek, short wait times, and easy accessibility of pharmacies within communities as facilitators of pharmacy-based care quality.

Providers expressed motivation to improve care, and considered it within their role and scope to provide comprehensive contraceptive counseling for AGYW, who they considered marginalized in their communities. Providers voiced specific concerns about the misuse or overuse of EC among marginalized AGYW, and wished to help them prevent pregnancy more effectively. Interviewees spoke of how some AGYW will come in for EC up to three times per week, and sometimes could not procure the money for the method prior to 72 h passing after unprotected intercourse. Providers spoke of feeling tension between dispensing EC frequently to AGYW to keep their business and trust, while also feeling an obligation to counsel them about other options that may be more effective in the longer term:

*“I thought that the P2[EC] is just an emergency pill not a method of family planning–now it’s like it’s the routine because the youths use it a lot. At times you feel like advising somebody: instead of using P2 twice or thrice a week, there are other methods available.” (Pharm tech, female, age 30–39)*

Suggested opportunities to advance the quality of contraceptive care included setting aside space for contraceptive counseling, providing formal training on contraceptive service delivery to pharmacy staff, improving staffing at peak hours, and the provision of various tools and job aids to pharmacy providers. Specifically, they named a need for training related to contraceptive side effects and how to counsel clients about both potential side effects and treatment of side effects of the various methods. When asked about whether they would find it helpful to be able to offer contraceptive decision support tools to clients, providers were generally enthusiastic, especially regarding potential mobile and digital health solutions:

*“I think that [digital contraceptive decision support tool] would be good because for the youth, that generation is digitally savvy, so they know what is happening. Because…they are always on TikTok or WhatsApp, so it would also help them when they can access an app and they know what [contraceptive method] to use. (Pharm tech, male, age 40–49)*

Providers had concerns about the impact of limited AGYW literacy in informal settlements, privacy while engaging with a tool, and the amount of time AGYW might be willing to spend “reading,” but felt that new tools for contraceptive decision support within the pharmacy setting would be beneficial to an underserved group of young clients and save provider time.

#### Flow mapping

**Fig. 1:**
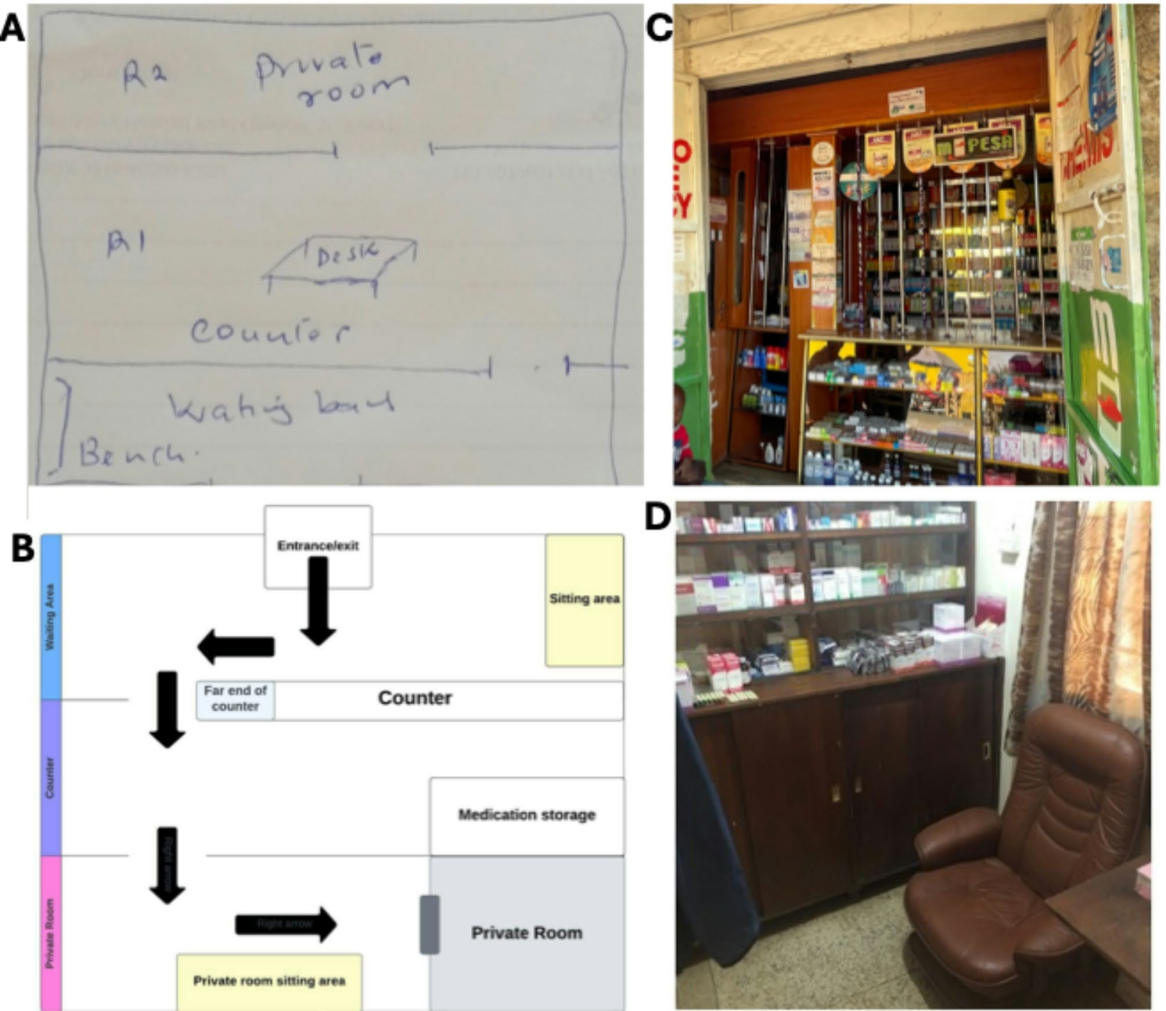
Schematic and images of pharmacy layout. **A** Sketched layout of study pharmacy **B **Typical layout of study pharmacies **C **Example of pharmacy counter **D**Example of pharmacy private consultation room

**Fig. 2:**
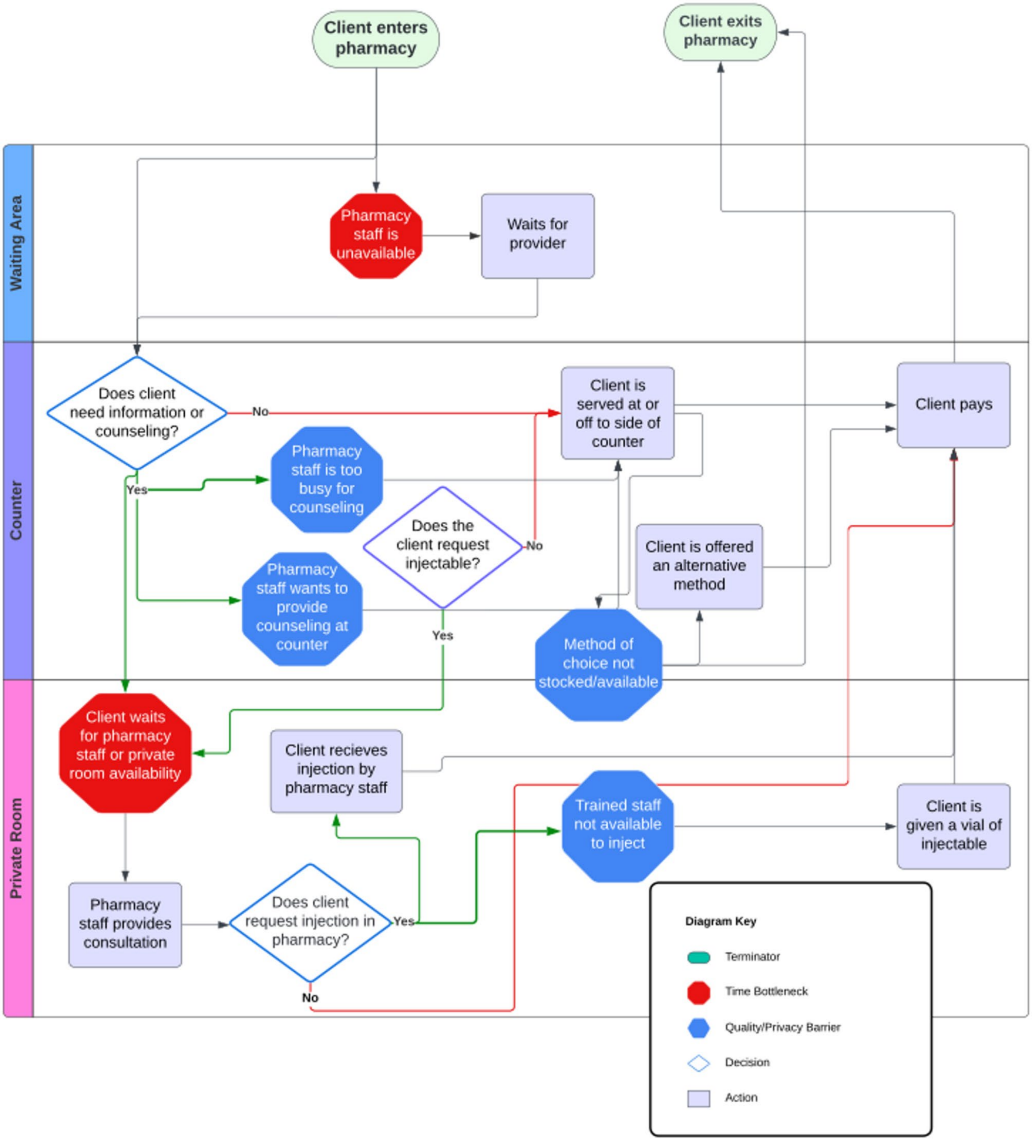
Flow map depicting overall process of pharmacy-based contraceptive service delivery

The sketched floor plans of the pharmacies were combined into a typical layout, shown in Fig. 1. A young woman starts the process of seeking/obtaining contraception in a pharmacy waiting area or at a counter window. Depending on the client’s request and space availability in the pharmacy, the client is served at the counter or in a private consultation room. All pharmacies included in the study had access to a private room or store room, which staff described using for patient counseling and injections. Pharmacy providers identified several time bottlenecks, shown in Fig. 2, beginning in the waiting area if a staff member is serving another client or there is a queue of patients. If the client has questions or needs counseling or consultation, they may need to wait until the pharmacy provider has finished serving other clients or until the consultation room is available. If the pharmacy is too busy, clients may not be given the option to wait for private consultation, and/or pharmacy providers may require all clients to be served at the counter or decline to provide counseling. If the client is seeking injectable contraception, there may be additional time spent waiting to be injected after purchasing the vial of medication, or waiting for a staff member trained to do injections. According to pharmacy providers, quality bottlenecks occur most frequently when there are multiple clients waiting to be served; AGYW may receive no contraceptive counseling or pharmacy staff may not have time to full address their questions. Providers also identified limited staff training, specifically related to counseling about and managing side effects, and method stock outs as bottlenecks hindering quality contraceptive care. When clients needed methods that were out of stock or not offered at that pharmacy, or their issue could otherwise not be managed, providers’ referral patterns differed widely and typically did not refer clients to a particular provider known to be youth friendly, but to nearby public sector clinics. Informal verbal referrals were the norm rather than written or digital referrals. Privacy bottlenecks were common, usually occurring whenever there were more than one client at the counter, or counseling was provided at the counter within earshot of other clients. Additionally, being seen by other clients going into and out of a consultation room could be experienced as an invasion of privacy for AGYW.

#### Mystery client experiences

Mystery clients conducted 20 unique pharmacy visits and obtained injectable contraception (*n* = 6 visits), OC (*n* = 9), EC (*n* = 6). At one visit, both injectable contraception and EC were purchased. Of note, mystery clients bought the vial of injectable contraception but did not request provider injection, which is a common scenario in the study setting. Mystery client visits took a median of 27 min for injectable contraception, 19 min for OC, and 25 min for EC; the overall range was 4–34 min for all methods. One pharmacy did not have injectable contraception currently in stock, and another only had the more expensive name-brand injectable and not the cheaper generic version, as it was sold out. Mystery clients rated perceived privacy as high at 13 visits, moderate at 2 visits, and unsatisfactory at 5 visits. At five visits, clients appreciated that providers packaged the purchased method in a discreet brown paper bag. Privacy was enhanced by being served in a private consultation room (4 visits) or being the only client at the counter (7 visits). One client noted,

*“When I got the opportunity to be served, as soon as I mentioned I wanted family planning services, she asked me to enter the pharmacy and showed me to the door to the consultation room where there was maximum privacy.” (Visit 16)*

Mystery clients also reported pain points where the encounter lacked privacy. One male pharmacist was noted to be in conversation with other male friends at the counter; even after the client requested privacy, he insisted on serving her at the counter where others could hear them. At some visits, the provider served the clients at the counter where other clients could hear:

*“At the time he was taking me through the methods there was no privacy since there was another client standing directly next to me at that time. I was not comfortable by how he offered the service.” (Visit 20)*

Pharmacy providers took the time to answer the mystery clients’ assigned method questions in 15 out of 20 visits, though the providers’ affect and perceived willingness to provide contraceptive information tailored to the client was highly variable. Mystery clients’ experiences of counseling ranged from friendly providers offering comprehensive counseling on all available methods or explaining the difference between four different OC options to nonexistent counseling with providers that seemed impatient or rushed:

*“[The pharmacist was] very polite and patient. She showed me all the family planning methods on a pictorial booklet. She answered all the questions as well. We discussed and decided on a method…” (Visit 5)*

*“Since she seemed in a hurry to get back to the phone, she wasn’t able to give me ample time to ask her questions for deeper understanding on the method. She only gave me brief information on when I should come back for the next injection.” (Visit 8)*

Mystery clients also provided notes on *how* their questions were answered, revealing several instances of inaccurate information. At 2 visits, providers recommended against use of injectable contraception due to the mystery client’s younger age and nulliparity. At one visit, the provider claimed that one must not use EC more than twice per year; at another, the provider said one must not use EC more than twice in one cycle. Another provider declined to answer the client’s question about side effects:

*“He didn’t talk about the side effects since he claimed that it could discourage someone from using the method. He proposed that in case I experienced side effects that I should feel free to go back.” (Visit 6)*

## Discussion

In this study, we triangulated data from interviews with pharmacy staff, flow mapping, and mystery client methods to explore barriers and facilitators to improving the quality of contraceptive care among AGYW in community pharmacies in Kenya. Our findings contribute new insights into the perspectives of pharmacy providers on their perceived roles in contraceptive service delivery and opportunities to improve care in conversation with mystery client experiences. While prior studies have examined pharmacy provider contraceptive knowledge, dispensing practices, and regulation [6, 15, 22], provider perspectives on quality of care have not been explored in detail. Additionally, to our knowledge, this is the first study to apply flow mapping to characterize bottlenecks affecting quality in pharmacy-based contraceptive service delivery.

The pharmacy providers interviewed in this study strongly agreed that the provision of quality contraceptive services, including counseling, should be within their scope of practice. In addition, providers expressed a strong ethical rationale for serving AGYW who need contraception, as well as empathy for the social stigma and life stressors they encounter in accessing SRH services. These findings align with several studies among young people in a variety of countries including Kenya, in which pharmacy provider approachability and non-judgment are contrasted with public sector providers’ attitudes towards youth and contraceptive limiting [1, 3, 4, 29]. Despite their motivation, pharmacy staff frequently acknowledged their limited training as a quality barrier, and instances of bias towards youth and restriction of contraceptive choices stood out in our study, consistent with related research highlighting inaccuracies in counseling (though this issue is not limited to the pharmacy setting) [16, 17]. Many pharmacy staff and mystery clients described providers going above and beyond to provide counseling to young people, and our prior qualitative interviews with AGYW around service delivery preferences uncovered a strong demand for information and counseling [30]. In a discrete choice experiment among 500 AGYW in Kisumu County aged 15–20, participants voiced a strong preference for face-to-face contraceptive counseling over a mobile phone-based intervention, though 52% of participants did not have access to a mobile phone or shared a phone with a family member [31]. These data support the need for pharmacy provider training and job aids to improve quality in line with AGYW preferences. That said, it is essential to acknowledge that for some AGYW, contraceptive care aligned with their preferences may mean barely speaking to the provider and receiving their method in a discreet brown paper bag in the briefest encounter possible in the attempt to avoid detection. While such care may not necessarily be considered high quality through the lens of the Bruce/Jain framework, it may be person-centered care from the perspective of young people. The principles of harm reduction [32] may help in conceptualizing how while perhaps not ideal, the low barrier access meets AGYW where they are and provides some protection from undesired pregnancy.

Our data spotlight several opportunities to improve quality of care. Pharmacy providers emphasized AGYW’s lack of contraceptive knowledge and non-evidence-based concerns about the harms of hormonal contraception– findings that are well corroborated in prior studies [33–35]. Given that young people are more likely to be new users and lack experience and exposure to health provider teaching, interventions to make person-centered contraceptive counseling available (but not required) in the pharmacy setting could have a high impact on supporting the informed choice and free choice elements of contraceptive autonomy [36]. Given the constraints on pharmacy staff time and their conflicts of interest as businesspeople, client-facing contraceptive decision support tools are needed in addition to job aids and training for providers. The intersecting time and privacy bottlenecks in pharmacy-based contraceptive service delivery for AGYW also represent key opportunities for improving care. Pharmacy providers explained how AGYW minimized time spent at the pharmacy due to social stigma and fear of being seen, overheard, or missed at home; in other words, time bottlenecks are associated with privacy bottlenecks. Mystery clients reported discomfort being served within earshot of others at the counter, and expressed relief and satisfaction when they were the only client at the counter or served in a private room. These bottlenecks, while intrinsic to the small community pharmacy setting and not eliminable, could be improved by systems-improvements implementation interventions targeting AGYW clients’ experience moving through the pharmacy space. Furthermore, while providers have valid concerns that frequent EC use may not be the most effective or economical pregnancy prevention method, we advocate that EC access is critical and should not be limited or condemned. Rather, providers could build rapport with clients over time and offer contraceptive decision support tools and counseling that allows AGYW to choose other methods that better meet their needs.

Our study’s strengths include methods triangulation, which fosters a more comprehensive and integrated understanding of provider and client stakeholder perspectives. Our use of flow mapping was novel, and was successful in identifying specific quality bottlenecks for emphasis in future implementation research and interventions. Data were collected and analyzed by an experienced, multi-disciplinary team grounded in the local context through lived experience. Our study’s generalizability is limited by our focus on a single western Kenyan county and the in-depth nature of our data collection requiring a small sample size; however our findings share similarities with studies in other geographic areas, such as coastal Kenya [3, 6], Nigeria [4], and Uganda [37, 38], suggesting relevance to understanding pharmacy-based quality of contraceptive care for youth in other settings. Another limitation is that we did not engage directly with AGYW seeking contraceptive care at the study pharmacies, choosing rather to collect data through trained mystery clients and draw on our prior in-depth work with youth and contraceptive preferences and decision-making [31, 34, 39] to inform our analysis. Since mystery clients did not receive injections as part of data collection, we were unable to include first-hand experiences of injectable contraceptive administration in the flow mapping data, limiting our ability to comment on associated time or privacy bottlenecks. Our insights should not be directly applied to smaller pharmacies or drug shops without a private room to accommodate counseling and injections.

Pharmacy-based contraceptive care is not a “panacea” for all adolescents and youth, as expressed in the 2023 commentary by Gonsalves et al. [39]: contraception options in pharmacies are typically limited, clients have to pay for services, and the counseling provided is variable and often low-quality. However, pharmacies continue to be preferred access sites for many AGYW, and our data suggest multiple avenues to improve contraceptive care quality through efforts to enhance privacy and counseling, with the potential for improved reach, person-centeredness, and ultimately reproductive health outcomes for youth. Future research is needed to find the balance between improving quality and maintaining the qualities that make pharmacies preferable in the first place (e.g., convenience, anonymity, speed of service) in various contexts. 

## Supplementary Information

Supplementary Material 1.

Supplementary Material 2.

## Data Availability

The anonymous dataset/transcripts used and/or analysed during the current study are available from the corresponding author on reasonable request.

## Abbreviations

AGYW: Adolescent girls and young women
LMICs: Low- and middle-income countries
OCs: Oral contraceptives
ECs: Emergency contraceptives
