# Supplementary material for: Exploring opportunities to enhance the quality of pharmacy-based contraceptive service delivery for adolescents and young women in Kenya: a multimethod qualitative study
Source: BMC Health Serv Res. 2025 Jun 3;25:790. doi: 10.1186/s12913-025-12933-0 (PMC12131450; doi:10.1186/s12913-025-12933-0)
Supplement: Supplementary file 1 — Supplementary Material 1. [file 12913_2025_12933_MOESM1_ESM.pdf]

MARA Pharmacy Study: Flow Mapping Guide

Ask the pharmacy staff member to lead you through the physical space of the pharmacy, from outside the door, to where clients wait, to where clients and pharmacy staff interact, to the stock room, to the register, to the payment process, etc.

Draw an annotated physical map below of each step a client and/or staff member goes through to receive/provide various contraceptive method. *What do you notice about the physical space? Look at the posters on the walls, price-lists, advertisements, etc. <u>Take written notes and write a brief report after departing the pharmacy.</u>*

Probe:

- Physical space: Describe. How many staff? Are there posters, advertisements, job aids on the walls that you notice?
- Provision of injectables – where is this done?
- How long do clients typically wait for various steps in the process?
    - How does time of day or day of the week affect waiting time and privacy?
- Privacy at various steps in the process
- Potential bottlenecks in contraceptive service delivery – what tends to make providing methods more challenging/take more time

Please use the below table to record 1-2 observed client observations per pharmacy:

Observation 1:		Observation 2:	
**Characteristic**	**Description**	**Characteristic**	**Description**
Approximate client age		Approximate client age	
Method desired:		Method desired:	
Cost of method		Cost of method	
What do you notice about client/pharmacist interactions?		What do you notice about client/pharmacist interactions?	
Notes on privacy		Notes on privacy	
Time-motion		*Time-motion*	
**Action**	**Number of minutes /Description**	**Action**	**Number of minutes /Description**
Waiting in queue		Waiting in queue	
Time talking to pharmacist		Time talking to pharmacist	
Waiting for method, location of waiting		Waiting for method, location of waiting	
Receiving method		Receiving method	
Paying for method		Paying for method	
Other		Other	
