# Supplementary material for: Exploring opportunities to enhance the quality of pharmacy-based contraceptive service delivery for adolescents and young women in Kenya: a multimethod qualitative study
Source: BMC Health Serv Res. 2025 Jun 3;25:790. doi: 10.1186/s12913-025-12933-0 (PMC12131450; doi:10.1186/s12913-025-12933-0)
Supplement: Supplementary file 2 — Supplementary Material 2. [file 12913_2025_12933_MOESM2_ESM.pdf]

MARA Pharmacy Study: Interview Guide

Thank you for being willing to speak with me about your experience providing contraceptive services in the pharmacy setting. I'd like to start with some basic questions about which methods are available and how they are dispensed. I want to remind you that this information is confidential and will not be linked to you or to this pharmacy. We are simply here to learn about the care that is offered – there are no right or wrong answers.

Provision of Contraceptive Methods, Part A

1. Which family planning methods are available at your pharmacy? *List methods.*

    a. Could you please tell me about your experience providing these family planning methods in the pharmacy?
    b. **Depo:** Could you say more about your experience selling Depo?
        i. Do you provide both intramuscular and subcutaneous (Sayana Press) forms? Who injects the depo?
        ii. When you provide depo, do you calculate a TCA date (date they need to come back for next injection)? If so, how do you do this (i.e., do they know the number of weeks until the TCA?)
        iii. Do your clients also come with their usual clinic cards? *If so,* how do you use these cards?
    c. **Implants:** Are contraceptive implants available in this pharmacy?
        i. [If yes] Which implant(s)?
            1. What do you think are the **advantages** of offering implant insertion/removal in your pharmacy? (e.g. keep clients, provide needed service, have the expertise)
            2. What are the **disadvantages**? (e.g. inadequate training, removal not available, takes too much time, need to sterilize equipment)
            3. Have you ever had any problems providing this care?
        ii. [If no] Are you aware of pharmacies offering implant insertion and/or removal in Kisumu? Could you say more about this?
            1. What do you think are some advantages and disadvantages of providing implants in the pharmacy setting? Why do you think that?

2. Does your pharmacy keep records on how many of each method are dispensed per week or per month? If yes:
    a. How many of each method are dispensed per week (or per month) at your pharmacy?
    b. Have you noticed any trends over time?

3. How much does each method cost? How much does a pregnancy test cost, if available?

4. Does your pharmacy record the dates of birth or ages of clients who come in for methods to prevent pregnancy? why or
    a. Why or why not?

Provision of Contraceptive Methods, Part B

5. Which methods are most popular among adolescent girls and young women? Why do you think this is?

6. Without mentioning any names, do any specific stories or interactions with young women clients requesting methods to prevent pregnancy come to mind?
    a. What are your interactions typically like with adolescents and young women who present for family planning? Why do you think that is?

7. Are there any requirements clients need to meet or questions they need to answer before receiving their desired method? What are these (probe for each method)?
    a. Do you assess the date of the last menstrual period or recommend a pregnancy test prior to dispensing certain methods?
        i. Which methods? Could you please explain how you use the LMP or pregnancy test to guide care, if at all?

8. Other than methods to prevent pregnancy, what are young women aged 15-24 most likely to request or purchase in the pharmacy? Probe: do young women request services to terminate their pregnancies? How frequently does this occur?

9. Does your pharmacy offer PrEP services?
    a. What connections do you see between offering PrEP and methods to prevent pregnancy to young women?

<u>Barriers and Facilitators to Providing Contraceptive Counseling</u>

1. Could you describe for me the types of questions adolescent girls and young women have about methods to prevent pregnancy when they are seeking care?

2. Do you think adolescent girls and young women experience any stigma when seeking family planning in the pharmacy setting? Why or why not?
    a. Probe: If a young girl frequents the pharmacy to buy condoms or P2, how do you think they feel purchasing these items?
    b. How common is it for men to buy P2 or pills?

3. What role do you think pharmacy staff have in advising clients on method choice?
    a. Why is that?
    b. What role would you like to have?

4. Are there any guidelines or policies you follow when providing methods to prevent pregnancy in the pharmacy setting?

5. Do you ever refer clients seeking family planning to another health facility? If yes, what are some of the reasons you might refer them? Which facility/facilities do you usually refer them?
    a. What would you do if a client requested a family planning method you do not have at the pharmacy?
    b. What would you do if a client complained of a serious side effect of a method?

6. What training have you had, if any, on family planning method options, characteristics, and side effects?
    a. If has had training, where did you get this training?
    b. What kind of training would you like to receive around family planning?

7. Privacy is often named as a reason for adolescent girls and young women to prefer pharmacies for contraceptive services. Could you comment on the level of privacy available at your pharmacy? For example, if a 17 year-old requests P2 (emergency contraception), would the person standing behind her in line know what she was requesting?
    a. What space is available to answer client questions about their methods? Can you think of a time when you answered questions? What did the client ask? How did you answer?

8. How would you like to see contraceptive counseling offered in the pharmacy, if at all?
    a. What effect do you think this would have on privacy?

9. Young people may benefit from knowing more about all their options – including effectiveness, side effects including bleeding changes, and how methods work. What do you think would be the best way to provide this information in the pharmacy setting, especially given that each girl will have different needs?

10. Given how busy pharmacy staff are, one idea would be to offer a tablet- or phone-app where adolescents and young women could use an app while in the pharmacy to help them learn about all their options.
    a. What do you think about this? Let's talk about how this could work (when should they use app, how long should it take, where would they use it in the pharmacy).

11. From your perspective, what are the main barriers, or challenges, to providing contraceptive counseling in the pharmacy setting?
    a. Probes:
        i. Space, pharmacist training, time, stigma, legal concerns, young women don't want to be counseled, pharmacists don't get paid for counseling, etc.

12. From your perspective, what are the main factors that would make it easy or possible to provide contraceptive counseling in the pharmacy setting?

13. What do you think could be done (in other words, programs or interventions) to improve the quality of the family planning services in pharmacies? Why?

Thank you very much for your time. This concludes the structured interview portion of the study; now I'd like to move on to the Flow Mapping questions.
